# Pyrene Carboxylate Ligand Based Coordination Polymers for Microwave-Assisted Solvent-Free Cyanosilylation of Aldehydes

**DOI:** 10.3390/molecules26041101

**Published:** 2021-02-19

**Authors:** Anirban Karmakar, Anup Paul, Elia Pantanetti Sabatini, M. Fátima C. Guedes da Silva, Armando J. L. Pombeiro

**Affiliations:** 1 Centro de Química Estrutural, Instituto Superior Técnico, Universidade de Lisboa, Av. Rovisco Pais, 1049-001 Lisbon, Portugal; kanupual@gmail.com (A.P.); e.pantanettisabatini@studenti.unicam.it (E.P.S.); fatima.guedes@tecnico.ulisboa.pt (M.F.C.G.d.S.); 2 Peoples’ Friendship University of Russia (RUDN University), 6 Miklukho-Maklaya Street, 117198 Moscow, Russia

**Keywords:** coordination polymer, crystal structure analysis, heterogeneous catalysis, microwave, solvent-free, cyanosilylation reaction

## Abstract

The new coordination polymers (CPs) [Zn(μ-1κ*O*^1^:1κ*O*^2^-L)(H_2_O)_2_]_n_·n(H_2_O) (**1**) and [Cd(μ_4_-1κ*O*^1^*O*^2^:2κ*N*:3,4κ*O*^3^-L)(H_2_O)]_n_·n(H_2_O) (**2**) are reported, being prepared by the solvothermal reactions of 5-{(pyren-4-ylmethyl)amino}isophthalic acid (H_2_L) with Zn(NO_3_)_2_.6H_2_O or Cd(NO_3_)_2_.4H_2_O, respectively. They were synthesized in a basic ethanolic medium or a DMF:H_2_O mixture, respectively. These compounds were characterized by single-crystal X-ray diffraction, FTIR spectroscopy, thermogravimetric and elemental analysis. The single-crystal X-ray diffraction analysis revealed that compound **1** is a one dimensional linear coordination polymer, whereas **2** presents a two dimensional network. In both compounds, the coordinating ligand (L^2−^) is twisted due to the rotation of the pyrene ring around the CH_2_-NH bond. In compound **1**, the Zn(II) metal ion has a tetrahedral geometry, whereas, in **2**, the dinuclear [Cd_2_(COO)_2_] moiety acts as a secondary building unit and the Cd(II) ion possesses a distorted octahedral geometry. Recently, several CPs have been explored for the cyanosilylation reaction under conventional conditions, but microwave-assisted cyanosilylation of aldehydes catalyzed by CPs has not yet been well studied. Thus, we have tested the solvent-free microwave-assisted cyanosilylation reactions of different aldehydes, with trimethylsilyl cyanide, using our synthesized compounds, which behave as highly active heterogeneous catalysts. The coordination polymer **1** is more effective than **2**, conceivably due to the higher Lewis acidity of the Zn(II) than the Cd(II) center and to a higher accessibility of the metal centers in the former framework. We have also checked the heterogeneity and recyclability of these coordination polymers, showing that they remain active at least after four recyclings.

## 1. Introduction

Coordination polymers are commonly viewed as a type of exceedingly popular functional materials because of their charming 1D, 2D and 3D architectures, in addition to their substantial utility in numerous applications, which include molecular sensing, gas storage and separation, etc. [1,2]. These materials can be deliberately designed to produce explicit structures, topologies and intriguing properties, which are of high demand [3].

Among various applications of the synthesized coordination polymers (CPs), catalysis is one of the most important and explored areas of research where they have been employed, often as heterogeneous catalysts [4,5,6]. This is largely due to their robustness and insoluble nature in most of the common organic solvents. The important organic transformations for which CPs have been developed and employed as heterogeneous catalysts include cyanosilylation of aldehydes or ketones [7], Knoevenagel condensation [8], oxidation of alkanes, alcohols or olefins [9], Henry reaction [10,11], ring-opening of epoxides [12], transesterification [13], cascade [14] reactions, etc.

Furthermore, in the domain of catalysis, cyanosilylation constitutes an important C-C bond formation reaction, leading to cyanohydrins, which are regarded as a class of compounds with interest in chemistry and biology, being broadly applied for the synthesis of plenty of vital compounds, such as α-hydroxy acids and aldehydes and β-amino alcohols [15]. Trimethylsilyl cyanide (TMSCN) is one of the mostly used reagents employed for the synthesis of cyanohydrins promoted by the presence of a Lewis acid or base catalyst, due to its facile use which proceeds via nucleophilic addition to carbonyl compounds to generate cyanohydrin trimethylsilyl ethers. Along these lines, the development of a powerful catalyst, which can effectively catalyze such a reaction is an important area of present-day research [16]. Although an array of complexes involving metal ions from d-, f- and p-blocks have been employed as catalysts for such a transformation [17,18,19], they mostly act in a homogeneous manner, and just a modest number of heterogeneous catalysts have been documented in this direction [20,21].

Other relevant features of a catalytic reaction concern the employed conditions. Although many of the catalytic reactions are being performed under conventional heating, there is an ever-increasing demand for an alternate energy source which will not only be cost-effective but also highly efficient and environmentally friendly. Microwave irradiation (MW) provides a notable technique which can take lesser time to perform a reaction, generate a better yield and selectivity, and is an ideal choice to look for. For a long time, our group has been utilizing this technique for carrying out many catalytic transformations [22]. Recently, various CPs have been explored for cyanosilylation of aldehydes under conventional conditions [23]; however, microwave-assisted cyanosilylation of aldehydes catalyzed by CPs has not yet been significantly studied. Thus, the exploration of appropriate CP based catalysts for the microwave-assisted solvent-free cyanosilylation of aldehydes is a topic of great interest.

In the current study, we synthesized the pro-ligand 5-{(pyren-4-ylmethyl)amino}isophthalic acid (H_2_L) and constructed the one-dimensional [Zn(μ-1κ*O*^1^:1κ*O*^2^-L)(H_2_O)_2_]_n_·n(H_2_O) (**1**) and the two-dimensional [Cd(μ_4_-1κ*O*^1^*O*^2^:2κ*N*:3,4κ*O*^3^-L)(H_2_O)]_n_·n(H_2_O) (**2**) coordination polymers. They have been characterized by FTIR, thermogravimetric, elemental, and single-crystal and powder X-ray diffraction analyses. The insolubility and existence of both Lewis acid and basic (amine) centers in these CPs makes them favorable materials for bifunctional heterogeneous catalysis. Thus, we tested their heterogeneous catalytic activity towards the solvent-free microwave-assisted cyanosilylation reaction of different aldehydes under mild conditions.

## 2. Results and Discussion

### 2.1. Synthesis and Characterization

**Figure 1:**
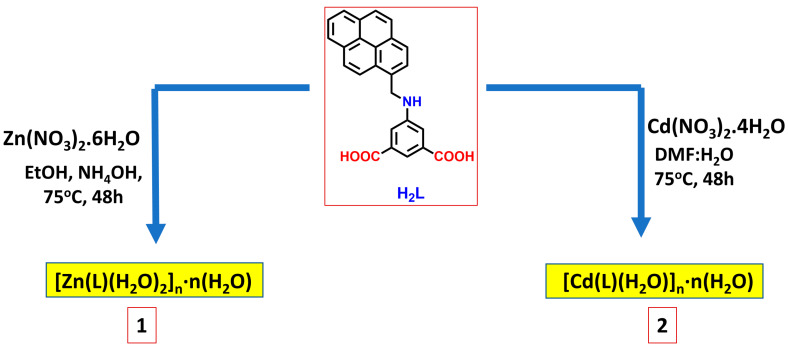
Synthesis of coordination polymers **1** and **2** by reactions of H_2_L and Zn(NO_3_)_2_·6H_2_O or Cd(NO_3_)_2_·4H_2_O, under solvothermal conditions.

The solvothermal reaction of H_2_L with Zn(NO_3_)_2_.6H_2_O in ethanol and NH_4_OH leads to the formation of the coordination polymer [Zn(μ-1κ*O*^1^:1κ*O*^2^-L)(H_2_O)_2_]_n_·n(H_2_O) (**1**) [L = 5-{(pyren-4-ylmethyl)amino}isophthalate]. Upon performing the solvothermal reaction using Cd(NO_3_)_2_.4H_2_O in the presence of DMF and water mixture, we obtained the coordination polymer [Cd(μ_4_-1κ*O*^1^*O*^2^:2κ*N*:3,4κ*O*^3^-L)(H_2_O)]_n_·n(H_2_O) (**2**) (Figure 1). In the FTIR spectra, the characteristic strong bands for the asymmetric stretching of coordinated carboxylate groups of **1** and **2** appear at 1640–1641 cm^−1^ and the symmetric stretching is observed at 1350–1370 cm^−1^. The C=C stretching frequencies of the aromatic rings are present in the 1561–1572 cm^−1^ and 1411–1421 cm^−1^ regions. The band attributed to the secondary amine group can be found in the 2914–2934 cm^−1^ region.

### 2.2. Thermogravimetric Analyses

**Figure 2:**
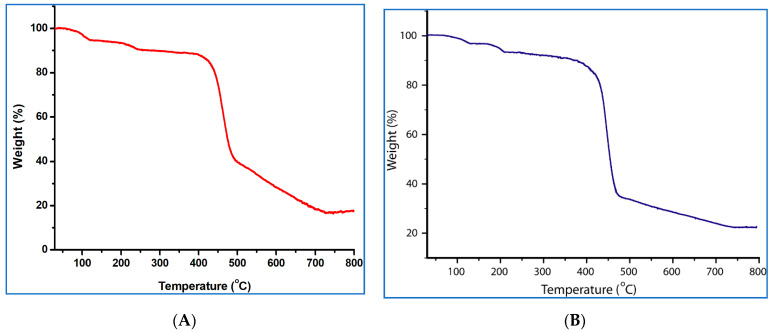
Thermogravimetric curves for **1** (**A**) and **2** (**B**).

Thermogravimetric analyses were performed under dinitrogen from room temperature to ca. 800 °C, with a heating rate of 5 °C min^−1^. Features of the thermal stability of the coordination polymers **1** and **2** are presented in Figure 2.

The coordination polymer **1** shows a total weight decrease of 10.1% between 59 and 263 °C, corresponding to the loss of the non-coordinated and coordinated water molecules (calcd: 10.5%). Afterwards it remains stable up to 388 °C and then starts to decompose until 739 °C (Figure 2A). Compound **2** shows a weight loss of 3.1% in the 65–135 °C temperature range, which is due to the removal of the non-coordinated water molecule (calcd: 3.5%). In the temperature range of 136–210 °C, it loses the coordinated water molecule, equivalent to a weight loss of 3.2% (calcd: 3.5%). However, above this temperature, the framework remains stable up to 364 °C and then starts to decompose until 745 °C (Figure 2B).

### 2.3. Crystal Structure Analysis

**Figure 3:**
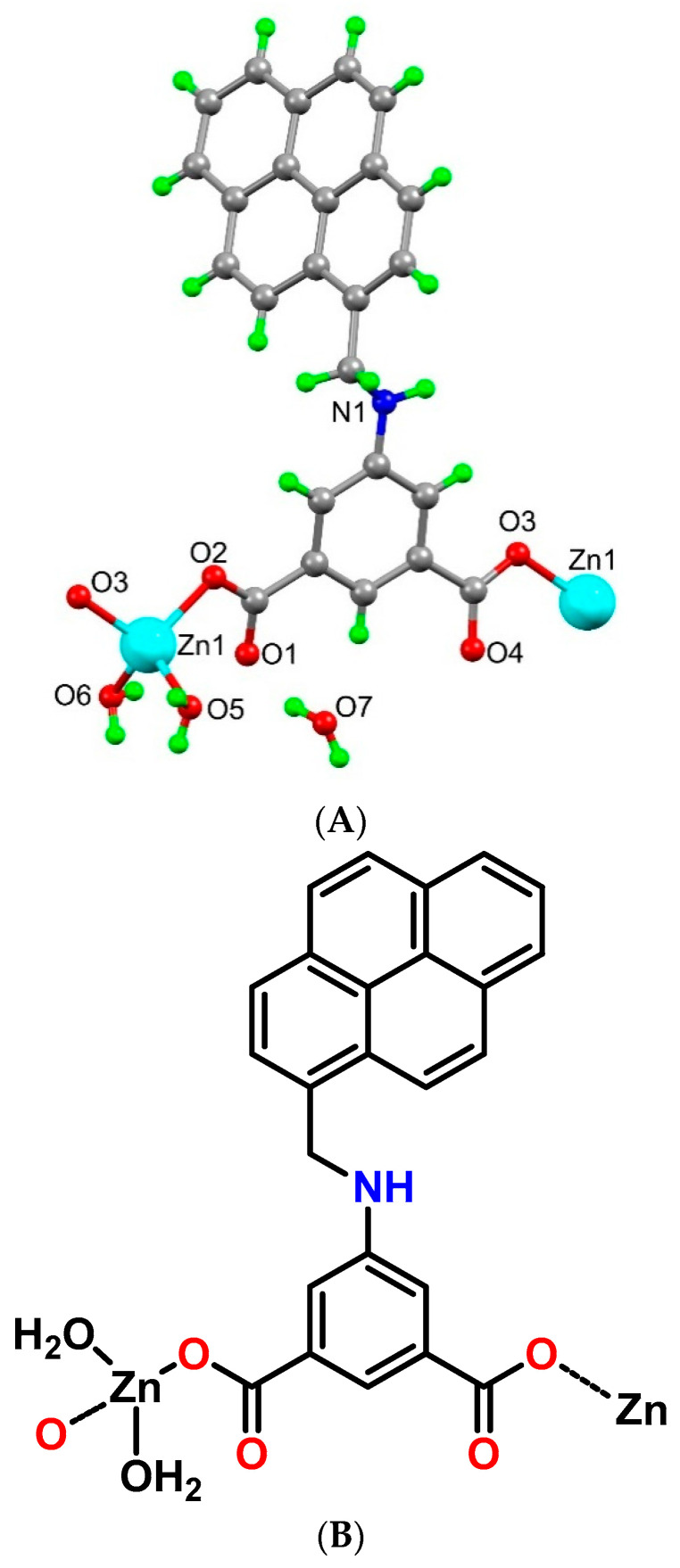

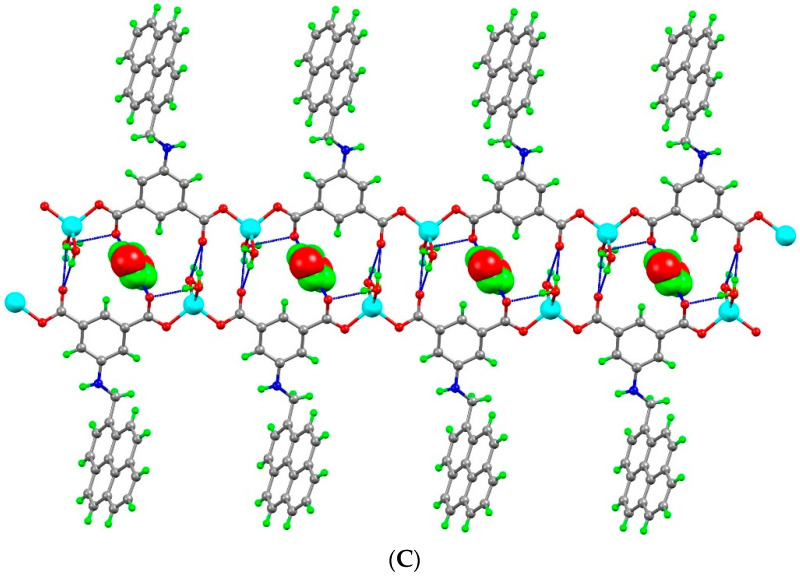
(**A**) Molecular structure of framework **1**. (**B**) Schematic representation of framework **1**. (**C**) Hydrogen bonded 1D chain of framework **1**.

The asymmetric unit of the coordination polymer **1** contains one zinc(II) ion, one L^2−^ ligand, two coordinated and one crystallization water molecules (Figure 3A,B). The Zn(II) center presents a distorted tetrahedral environment (τ^4^ = 0.91) [24], and via the deprotonated L^2−^ ligand, it generates a 1D polymeric chain (Figure 3C). The metal ion binds two carboxylate oxygen atoms from two L^2−^ units [Zn1-O2 2.007(17) Å and Zn1-O3 1.970(16) Å] and two water molecules [Zn1-O5 2.020(2) Å and Zn1-O6 2.025(2) Å]; the O–Zn–O bond angles fall within the range of 103.0(7)° to 119.7(9)°. The pyrene and isophthalate ring of the ligand are almost perpendicular with a dihedral angle of 73.34°. The observed non-planarity of the ligand is due to the relative twisting of the CH_2_-NH group, and the C4-N1-C9-C10 torsional angle is 163.21°. The carboxylate groups, working in a monodentate fashion, are nearly in the plane of the isophthalate ring (dihedral angles of 8.53° and 12.92°). The distance between two symmetry related Zn(II) ions in a chain is 10.239 Å, which is considerably larger than the shortest intermolecular distance of 5.412 Å between two metal ions in vicinal chains.

The 1D chains of coordination polymer **1** are hydrogen bonded through several O-H····O interactions between the coordinated water molecules and the carboxylate-O atoms, forming a two-dimensional hydrogen bonded network. Moreover, these 2D hydrogen bonded networks are further connected via π····π interactions (between pyrene rings) with a distance of 3.489 Å (Supplementary Materials Figure S1A), N-H····π interactions (between amine NH and pyrene ring, d_D-π_ 3.644 Å, Supplementary Materials Figure S1B) and C-H····π interactions (between pyrene CH and pyrene ring, d_D-π_ 3.451 Å), which expand the structure to the third dimension.

**Figure 4:**
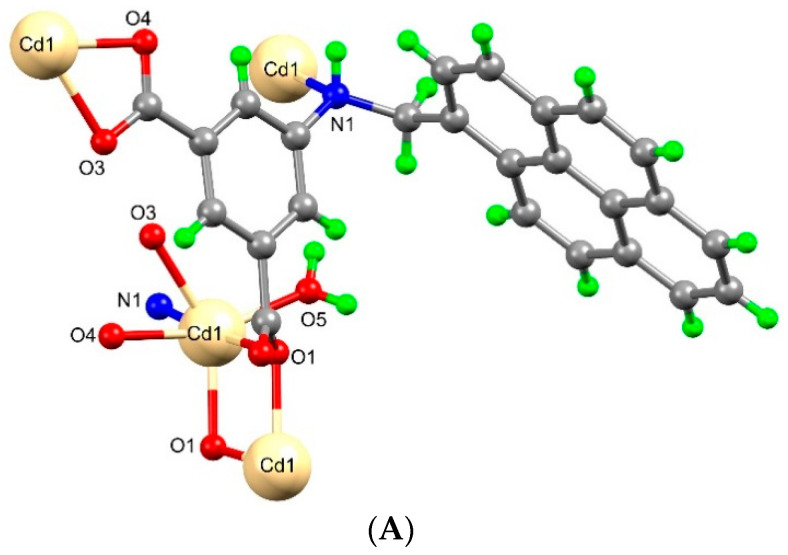

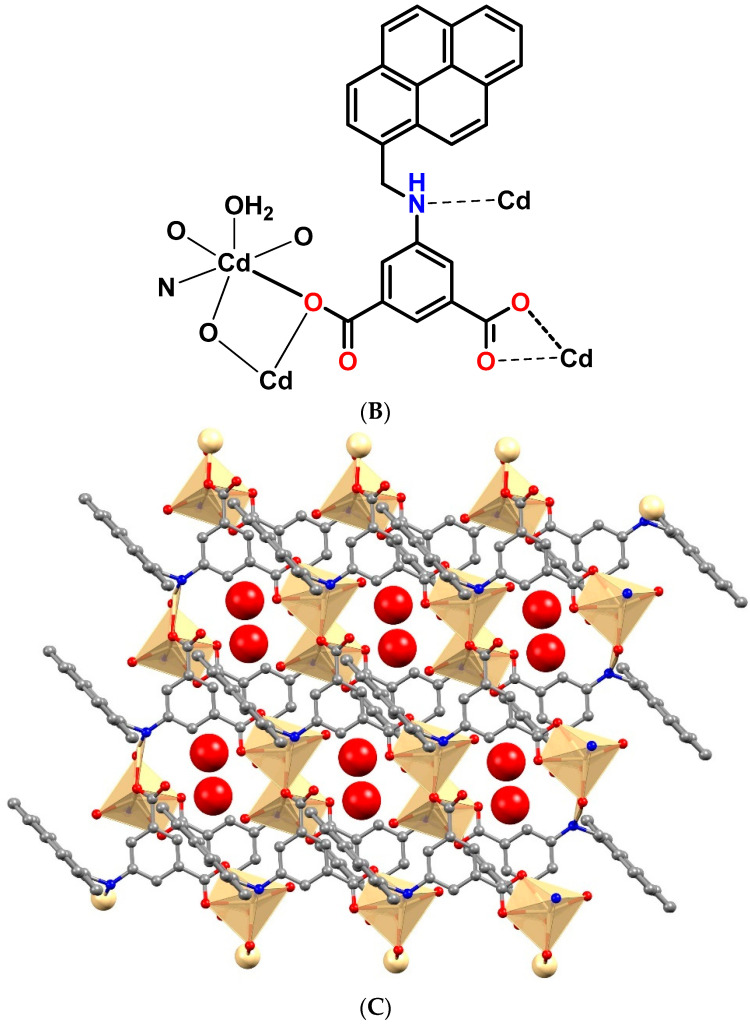
(**A**) Molecular structure of framework **2**. (**B**) Schematic representation of framework **2**. (**C**) Two-dimensional structure of framework **2** (the hydrogen-bonded water molecules are indicated as red balls).

The asymmetric unit of [Cd(μ_4_-1κ*O*^1^*O*^2^:2κ*N*:3,4κ*O*^3^-L)(H_2_O)]_n_·n(H_2_O) (**2**) contains one Cd(II) ion, one L^2−^ ligand and one of each coordinated and non-coordinated water molecules (Figure 4A,B). The distorted octahedral coordination environment of Cd(II) ion is occupied by four carboxylate-O from three adjacent L^2−^ ligands [Cd1-O1 2.228(2), Cd1-O1′ 2.451(3), Cd1-O3 2.343(2), Cd1-O4 2.401(3)] and one amine NH from a L^2−^ ligand [Cd1-N1 2.470(3)], and the remaining site is engaged with the water molecule [Cd1-O5 2.190(3)]. The O–Cd–O bond angles are within the range of 54.45(9)° to 173.60(9)°. In this coordination polymer, the amine NH participates in the metal coordination, which we have not observed in other coordination polymers. The deprotonated L^2−^ ligand coordinates simultaneously to four metal ions, with two of them linked by a bridging-chelate carboxylate group, another one where carboxylate is in a chelate-monodentate mode, and the remaining one by the amine NH group.

As in **1**, the organic ligand in **2** is highly twisted. The least-squares plane of the isophthalate ring makes a dihedral angle of 84.51° with the pyrene ring and one of the carboxylate groups is 40.17° twisted relatively to the plane of the aromatic ring it is attached to. The crystallographically related Cd-ions are arranged in a dinuclear [Cd_2_(COO)_2_] cluster forming a secondary building block unit. The Cd····Cd distance within the dinuclear clusters is of 3.695 Å. Assembly of six L^2−^ ligands and the dinuclear [Cd_2_(COO)_2_] cluster results the construction of a two dimensional framework (Figure 4C). Moreover, the coordinated and non-coordinated water molecules are hydrogen bonded with carboxyl groups of the L^2−^ ligand through O-H····O interactions. The amine NH is also engaged in a hydrogen-bonding interaction with carboxylate-O via N-H····O interaction.

### 2.4. Catalytic Activity Studies

**Scheme 1:**
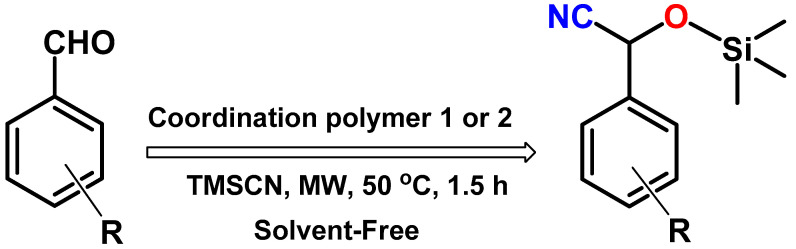
Aldehyde cyanosilylation reaction with trimethylsilyl cyanide (TMSCN).

Taking advantage of the high thermal stability and insolubility in most of the common organic solvents of the coordination polymers **1** and **2**, we tested them as heterogeneous catalysts for the cyanosilylation reaction of different aldehydes under microwave-assisted solvent-free conditions (Scheme 1). To begin with, benzaldehyde was taken as a model substrate, trimethylsilyl cyanide (TMSCN) as the silylating agent and the CP **1** or **2** as catalyst, and various reaction conditions like solvent, temperature and catalyst amount, were optimized.

Under optimized conditions, a combination of benzaldehyde (0.105 g, 1.0 mmol), trimethylsilyl cyanide (250 µL, 2 mmol) and catalyst (10.2 mg for **1** and 10.8 mg of **2**; 2.0 mol%) was placed in a Pyrex tube covered with a Teflon cap and stirred at 50 °C, under microwave irradiation (5 W), for 1.5 h, under solvent-free conditions. The desired product was identified by ^1^H NMR spectroscopy (Supplementary Materials Figure S4), and the product yield was calculated, using the same procedure as reported in the literature [21].

**Figure 5:**
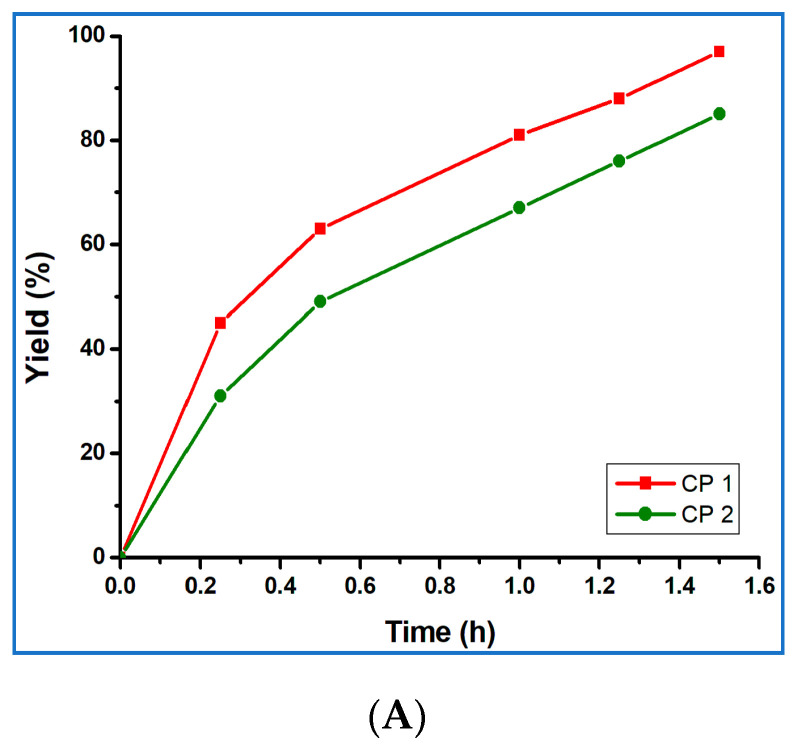

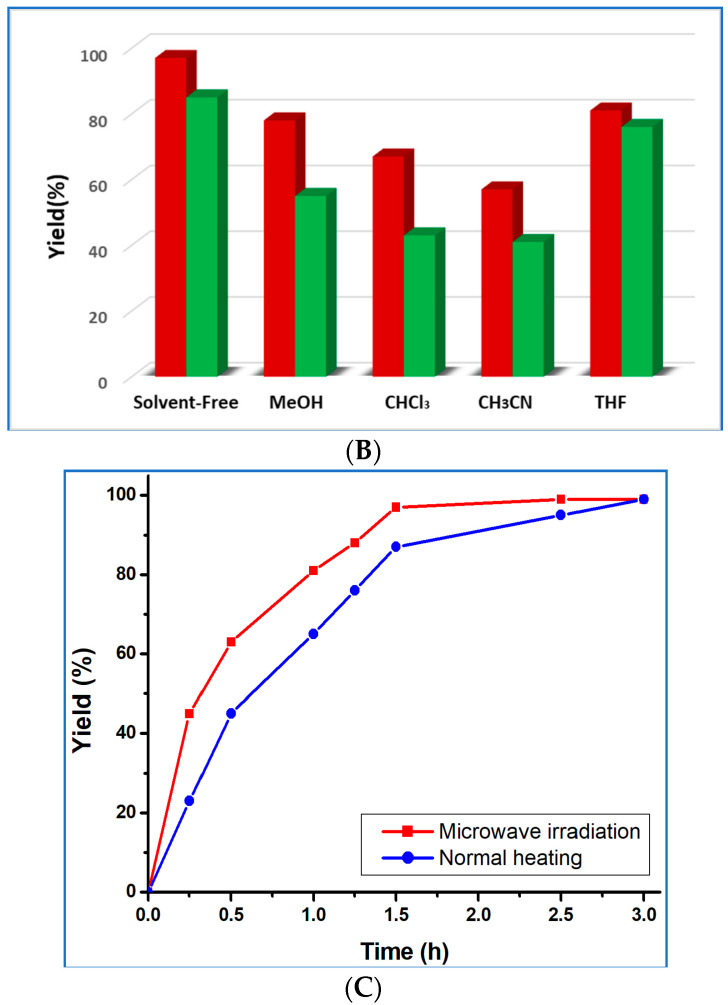
(**A**) Plot of yield vs. time for the microwave-assisted solvent-free cyanosilylation reaction of benzaldehyde, catalyzed by **1** or **2**. (**B**) Yield of cyanosilylation of benzaldehyde in different solvents catalyzed by compounds **1** (red pillar) and **2** (green pillar). (**C**) Plots of yield vs. time for the microwave-assisted (red line) and normal-heating (blue line) solvent-free cyanosilylation reaction of benzaldehyde, catalyzed by **1**.

We carried out the reactions in the presence of different solvents, such as MeOH, CHCl_3_, CH_3_CN or THF, and in the absence of any solvent. Solvent-free conditions show the maximum catalytic activity, leading to a product yield of 97% or 85% for coordination polymer **1** or **2**, respectively (entries 1 and 2, Table 1). The use of other solvents leads to lower yields in the range of 57–81% for **1** (entries 3–6, Table 1 and Figure 5B) and 41–76% for **2** (entries 7–10, Table 1 and Figure 5B). Performing the reaction in CH_3_CN gives the lowest yield of 57% or 41% for compound **1** or **2**, respectively. These observations are consistent with the competition of the solvent with the aldehyde substrate for the coordination to the metal.

In order to optimize the catalyst loading, we changed the catalyst amount from 1.0 to 2.0 mol% and observed a high enhancement of the product yield from 52 to 97% for **1** (entries 11 and 1, Table 1) or from 30 to 85% for **2** (entries 13 and 2, Table 1), but an additional increase of that amount (up to 5%) resulted only in an almost undetected yield improvement for **1** (entry 12, Table 1) and in no yield change for **2** (entry 14, Table 1). Thus, the optimal catalyst amount was considered as 2 mol% (relative to the aldehyde).

We also carried out the cyanosilylation reaction at different temperatures, to examine the effect of temperature. We obtained only 32% or 25% of product yield upon performing the reaction at room temperature (25 °C) (entry 15 or 18, Table 1), using **1** or **2** as catalyst, respectively. Increasing the reaction temperature from 25 to 50 °C results in an yield increase to 97% or 85% for **1** or **2** (entry 1 or 2), respectively. Further increase in the temperature to 60 °C did not improve the reaction yield (entries 17 and 20, Table 1).

A blank test was performed with benzaldehyde and TMSCN without any catalyst and resulted in only 10% yield of the final product after 1.5 h (entry 21, Table 1). We also checked the cyanosilylation reaction with zinc nitrate, cadmium nitrate and the free ligand (H_2_L) and the obtained reaction yields are within 18–23% (entries 22–24, Table 1). Thus, the optimized reaction conditions for the cyanosilylation of the aldehyde with TMSCN concern 2 mol% of the coordination polymer catalyst at 50 °C without any solvent. Under the optimized reaction conditions our coordination polymers **1** and **2** produce 97% and 85% of final product yields after 1.5 h under microwave irradiation, respectively. They correspond to a turnover number (TON) of 48.5 and 42.5, and a turnover frequency (TOF) of 32.3 and 28.3 h^−1^.

After further spreading the reaction time to 2.5 h, the final product yield increased up to 99% in the case of **1** or 87% for compound **2**, but the TOF value decreases markedly (from 32.3 h^−1^ to 19.4 h^−1^ for **1** and from 28.3 h^−1^ to 17.0 h^−1^ for **2**). The yield vs. time plots for the solvent-free cyanosilylation reaction of benzaldehyde and TMSCN catalyzed by **1** or **2** is presented in Figure 5A. We also performed the same reaction under normal heating conditions, instead of microwave, and the product yield was only 87% for compound **1** after 1.5 h, and it took 3 h to reach 99% (Figure 5B). Thus, the use of microwave irradiation accelerated the reaction quite significantly.

We also tested the activity of our catalysts **1** and **2** towards different substituted aromatic aldehydes. The aromatic aldehydes containing strong electron-withdrawing groups, such as nitro, bromo and chloro, exhibit the highest yields in the range of 95–99% for **1** and 89–96% for **2** (entries 1–4, Table 2). In contrast, the aldehydes containing electron-donating substituent, such as methyl or methoxy, display lower yields 58–75% (entries 7 and 8, Table 2). The *meta*- and *para*-hydroxy substituted aldehydes show moderate reactivity and produce the corresponding cyanohydrin derivatives within the yield range of 67–91% (entries 5 and 6, Table 2), being also above the corresponding yields for the aldehydes with eletron-donor substituents. These behaviors are in accord with the expected effect of the substituent on the electrophilic character of the aldehyde CHO carbon to undergo attack by the cyano group of TMSCN.

**Figure 6:**
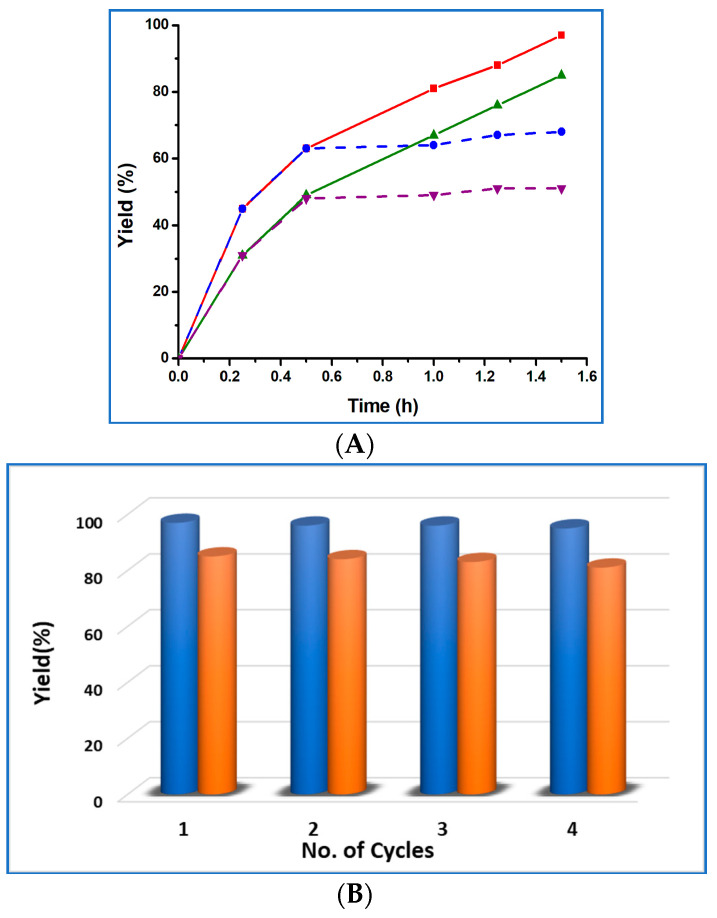
(**A**) Plots of product yield vs. time for the solvent-free cyanosilylation reaction, catalyzed by **1** (purple line) and **2** (red line) (blue dotted line indicates the catalyst **1** removal after 0.5 h and the green dotted line concerns the catalyst **2** removal after 0.5 h). (**B**) Effect of the catalyst recycling on the final product yield (blue pillars for compound **1** and brown pillars for compound **2**).

The recyclability of the catalysts **1** and **2** was also tested. At the end of a reaction cycle, the catalyst was separated by centrifugation, washed with CH_2_Cl_2_ and dried before recycling it. It could be successfully recycled at least for four consecutive cycles, without considerably losing its activity, as shown in Figure 6B.

To check the leaching and heterogeneity of our catalysts, we separated the catalyst by centrifugation after 0.5 h and kept the catalyst-free reaction mixture under the same environment for 1 h more. After elimination of the solid catalyst **1** or **2** from the reaction mixture, no perceptible rise in the yield was observed (Figure 6A, blue and green dotted lines), which substantiates the absence of leaching and the heterogeneous nature of the catalyst. FTIR and powder X-ray diffraction analyses of the catalyst before and after the catalysis reaction were performed, to check the structural integrity of our catalysts **1** and **2,** and no significant changes in their patterns were observed, as shown in Supplementary Materials Figure S2 and S3. These results support that the structures of the catalysts **1** and **2** remain intact after the catalytic reaction.

A comparison of the catalytic activity, towards the cyanosilylation of aldehydes with TMSCN, of our catalyst **1** with other reported coordination polymers is shown in Table 3. Our catalyst appears to be more efficient for such a reaction. For example, the cyanosilylation reaction of benzaldehyde and TMSCN at 50 °C, under solvent-free conditions catalyzed by [Pr(3,5-DSB)(Phen)]_n_ produce a yield of 78% after 3 h reaction time (entry 2, Table 3) [25]. The heterometallic CP [{Co_0.6_Ni_1.4_(H_2_O)_2_(Bpe)_2_}(V_4_O_12_)]·4H_2_O·Bpe gives a yield of 77%, at 50 °C, for 16 h, under solvent-free conditions (entry 3, Table 3) [26]. Another heterometallic coordination polymer, [NaCu(2,4-HPdc)(2,4-Pdc)], catalyzes the same reaction also under solvent-free conditions, for 24 h reaction time, producing an yield of 85% (entry 4, Table 3) [27]. Moreover, the mononuclear Sn(II) complex of 3-amino-2-pyrazinecarboxylate gives a reaction yield of 75%, at 50 °C, after 4 h (entry 5, Table 3) [28]. In this context, our catalyst **1** also leads to a 97% yield under solvent-free condition, at 50 °C, after 1.5 h reaction time (entry 1, Table 3). Therefore, our catalyst produces a higher product yield, in a shorter time, under solvent-free conditions.

Moreover, our catalyst **1** also shows a high efficiency in comparison to other reported coordination polymers, upon performing the cyanosilylation of benzaldehyde with TMSCN in the presence of a solvent (yield of 81%, in THF at 50 °C after 1.5 h, entry 6, Table 3). For example, the dinuclear Pb(II)-3-amino-2-pyrazinecarboxylate complex produces the reaction yield of 97%, at 50 °C, in DCM, after 6 h, a higher reaction time than our catalyst (entry 7, Table 3) [29]. An overall reaction yield of 57% after 48 h at 40 °C was obtained by using the 3D [Cu_3_(benzenetricarboxylate)_2_]_n_ MOF as catalyst (entry 8, Table 3) [30]. The 3D coordination polymer [Cd_2_(1,4-NDC)_2_(DMF)_2_]_n_ catalyzes the cyanosilylation of 4-nitrobenzaldehyde with an overall yield of 49% after 72 h at 50 °C (entry 9, Table 3) [31]. Moreover, the Zn(II) coordination polymers {[Zn(dpe)(μ-OOCCH_3_)_2_](H_2_O)}_n_ and {[Zn_3_(4,4′-bpy)_4_(μ-O_2_CCH_2_CH_3_)_4_](ClO_4_)_2_(4,4′-bpy)_2_(H_2_O)_4_}_n_ produce an yield of 14% and 22% upon performing the reaction in DCM, at room temperature, after 24 h, respectively (entries 10 and 11, Table 3) [32,33].

Based on our previous reports, a similar plausible catalytic reaction mechanism is proposed, wherein a Zn(II) center activates the aldehyde carbonyl group and trimethylsilyl cyanide with promotion of the nucleophilic attack of the CN group to the carbonyl carbon, leading to the construction of a C-C bond and thus to the cyanohydrin.

## 3. Materials and Methods

The synthesis of the ligand and coordination polymers were performed at a relatively high temperature. The chemicals were used as received from commercial sources. Bruker Vertex 70 instrument was used to record the FTIR spectra (4000–400 cm^−1^) in KBr pellets. The ^1^H NMR spectra for the ligand were recorded at room temperature, on a Bruker Avance II + 300 (UltraShield^TM^Magnet) spectrometer, operating at 300.130 MHz, and the chemical shifts are reported in ppm, using tetramethylsilane as the internal reference. TGA was carried out with a Perkin-Elmer Instrument system (STA6000), at a heating rate of 5 °C min^−1^, under a dinitrogen atmosphere. C, H and N elemental analyses for the coordination polymers were carried out by the Microanalytical Service of the Instituto Superior Técnico. A D8 Advance Bruker AXS (Bragg Brentano geometry) diffractometer, with Cu-radiation (Cu Kα, λ = 1.5406 Å), was used to collect powder X-ray data (PXRD), operated at 40 kV and 40 mA. The typical data-collection range was between 5° and 40°, and a flat plate configuration was used.

### 3.1. Synthesis of 5-{(pyren-4-ylmethyl)amino}isophthalic Acid (H_2_L)

The pro-ligand H_2_L was synthesized, using a two-step reaction. In the first step, 1-pyrenecarboxaldehyde (0.576 g, 2.5 mmol) and 5-aminoisophtalic acid (0.453 g, 2.5 mmol) were placed in a round bottom flask, and then dry methanol (25 mL) was added. After stirring overnight at room temperature, a yellow suspension was obtained. The solution was filtered, and the yellow solid was collected on filter paper, which was further washed several times, using fresh methanol. The obtained compound was dried in air and used in next step, without purification.

In the second step, the isolated compound (1.58 g, 4 mmol) was dissolved in 25 mL of methanol, and NaBH_4_ was gradually added to the suspension, until a colorless solution was obtained. Afterwards, the reaction mixture was left, stirring overnight, at room temperature. Upon completion, the methanol was evaporated, and 10 mL water was added into it, followed by the acidification until pH = 2, with a diluted solution of HCl. The obtained white solid product H_2_L was isolated by filtration and thoroughly washed with water. Yield: 89%. FTIR (KBr, cm^−1^): 3843 (wbr), 3381 (s), 3043 (m), 2873 (m), 2590 (m), 2509 (m), 2360 (w), 1932 (w), 1681 (s, ν_asym_ C=O), 1600 (s, ν C=C), 1542 (s, ν C=C), 1504 (s, ν C=C), 1425 (s, ν_sym_ C=O), 1330 (s), 1271 (s), 1244 (s), 1137 (m), 1083 (m), 997 (w), 964 (w), 950 (w), 889 (w), 844 (s), 759 (s), 711 (s), 694 (s); ^1^H-NMR (DMSO-d^6^): δ 8.48–8.45 (1H, m, Ar-H), 8.31–8.26 (4H, m, Ar-H), 8.25–8.15 (4H, m, Ar-H), 7.74 (1H, s, Ar-H), 7.43 (2H, s, Ar-H), 6.78 (1H, s, N-H), 5.05 (2H, s, -CH_2_). Anal. Calcd. for C_25_H_19_NO_4_ (M = 397.42): C, 75.55; H, 4.82; N, 3.52. Found: C, 75.52; H, 4.65; N, 3.61.

### 3.2. Synthesis of the Coordination Polymer [Zn(L)(H_2_O)_2_]_n_·n(H_2_O) (***1***)

A mixture of H_2_L (10 mg, 0.025 mmol) and Zn(NO_3_)_2_.6H_2_O (7.4 mg, 0.025 mmol) in 2 mL EtOH was prepared, and afterwards, 0.5 mL of NH_4_OH (30% aqueous solution) was added. This mixture was then put into an 8 mL glass vessel, sealed and heated at 75 °C, for 48 h. Then the reaction mixture was cooled to room temperature, giving colorless crystals of **1**. FTIR (KBr, cm^−1^) 3381 (mbr), 2914 (w), 1640 (s, ν_asym_ C=O), 1561 (s, ν C=C), 1504 (w), 1421 (s, ν C=C), 1352 (s, ν_sym_ C=O), 1322 (m), 1274 (m), 1199 (s), 1109 (w), 1076 (w), 979 (w), 846 (s), 828 (s), 781 (m), 712 (w). Anal. Calcd. for C_25_H_21_NO_7_Zn (M = 512.80): C, 58.55; H, 4.13; N, 2.73. Found: C, 58.23; H, 3.86; N, 2.42.

### 3.3. Synthesis of the Coordination Polymer [Cd(L)(H_2_O)]_n_·n(H_2_O) (***2***)

Cd(NO_3_)_2_·6H_2_O (8 mg, 0.025 mmol) and H_2_L (10 mg, 0.025 mmol) were dissolved in 2 mL of DMF:H_2_O (2:1, v/v) mixture, and the obtained system was sealed in a capped glass vessel and heated to 75 °C, for 48 h. The mixture was then cooled gradually, and colorless crystals of the coordination polymer **2** were obtained. FTIR (KBr, cm^−1^) 3320 (mbr), 2934 (w), 1641 (s, ν_asym_ C=O), 1572 (s, ν C=C), 1531(s), 1411 (s, ν C=C), 1370 (s, ν_sym_ C=O), 1284 (s), 1148 (w), 1080 (w), 1106 (m), 906 (w), 840 (s), 802 (s), 779 (s), 673 (m). Anal. Calcd. for C_25_H_17_CdNO_6_ (M = 539.82): C, 55.62; H, 3.17; N, 2.59. Found: C, 55.33; H, 3.15; N, 2.78.

### 3.4. Procedure for the Cyanosilylation Reaction of Aldehydes

A mixture of an aromatic aldehyde (1 mmol), trimethylsilyl cyanide (250 µL, 2 mmol) and 2.0 mol% catalyst (10.2 mg of **1** or 10.8 mg of **2**; relative to the aldehyde) was placed in a Pyrex tube. The tube was then put in the microwave reactor, and the reaction mixture was left, stirring under MW irradiation (5 W), at 50 °C, in solvent-free conditions, for the desired time. The catalyst was then separated by centrifugation, and the reaction mixture was evaporated, using a rotary evaporator. Then the final product was dissolved in CDCl_3_ and analyzed by ^1^H NMR (Supplementary Materials Figure S4).

### 3.5. Crystal Structure Determinations

X-ray quality single crystals of the coordination polymers **1** and **2** were placed in cryo-oil and mounted in a nylon loop, and the data collection was done by using a Bruker APEX-II PHOTON 100 diffractometer with graphite monochromated Mo-Kα (λ 0.71069) radiation, at room temperature. Phi and omega scans (0.5° per frame) were used for the data collection, and a full sphere of data was obtained. Bruker SMART [34] software was used to obtain cell parameters and refined the data by using Bruker SAINT [34] on all the observed reflections. By using the SADABS [35] program, the absorption corrections were performed. The crystal structures were solved by using the SIR 97 program and refined with SHELXL-2014/6 [36]. Calculations were performed, using the WinGX System-Version 2014.1 [37]. The H-atoms attached to C- and N-atoms were introduced at geometrically calculated positions and encompassed in the refinement, using the riding-model approximation; U_iso_(H) were defined as 1.2U_eq_ of the parent atoms for phenyl and 1.5U_eq_ of the parent atoms for the O- and N-atoms. Least-square refinements with anisotropic thermal motion parameters for all the non-hydrogen atoms and isotropic ones for the residual atoms were employed. In Supplementary Materials Table S1, the crystallographic data are summarized; in Supplementary Materials Table S2, selected bond distances and angles are presented; and the hydrogen bonding interactions are presented in Supplementary Materials Table S3. The Cambridge Crystallographic Data Centre (CCDC) codes 2057313 and 2057314 contain the supplementary crystallographic data for this paper. These data can be obtained free of charge from The Cambridge Crystallographic Data Centre.

## 4. Conclusions

We synthesized and characterized two new coordination polymers, namely [Zn(μ-1κ*O*^1^:1κ*O*^2^-L)(H_2_O)_2_]_n_·n(H_2_O) (**1**) and [Cd(μ_4_-1κ*O*^1^*O*^2^:2κ*N*:3,4κ*O*^3^-L)(H_2_O)]_n_·n(H_2_O) (**2**), constructed from 5-{(pyren-4-ylmethyl)amino}isophthalic acid (H_2_L) with Zn(NO_3_)_2_.6H_2_O and Cd(NO_3_)_2_.4H_2_O, respectively, under solvothermal reaction conditions. Single-crystal X-ray diffraction analyses revealed that the coordination polymer **1** features a one-dimensional linear structure, whereas **2** presents a two-dimensional network. The presence of Lewis acid (metal ion) and basic (amine group) centers, as well as the insolubility in organic solvents, makes these CPs suitable for heterogeneous catalysis.

Thus, we tested the catalytic activities of these synthesized coordination polymers towards the cyanosilylation reaction of various aldehydes with TMSCN, under microwave irradiation. They show a high activity for such a reaction under solvent-free conditions, mainly the Zn(II)-coordination polymer (**1**), which is more active than the Cd(II) one (**2**), what may reflect the higher Lewis acidity of the Zn(II) center in the former than that of the Cd(II) site in the latter. However, the simpler 1D nature of **1** with more accessible metal centers, in comparison with the two-dimensional coordination polymer **2**, can be another reason behind the higher catalytic efficiency of **1**. We also demonstrated the heterogeneous nature, the stability and recyclability of the catalysts **1** and **2**. They can be recycled at least four times, without losing activity appreciably. Moreover, the use of microwave irradiation in such a reaction reduces the reaction time significantly. This work provides further indication that coordination polymers constructed from simple ligands, bearing both Lewis acid and basic centers, can be applied as efficient heterogeneous catalysts in important types of reactions, such as that investigated herein.

## Figures and Tables

**Table 1:** Cyanosilylation of benzaldehyde with trimethylsilyl cyanide (TMSCN) with coordination polymer 1 or 2 as catalyst ^a^.

Entry	Catalyst	Temperature (°C)	Catalyst Amount (mol%)	Solvent	Yield ^b^ (%)
1	**1**	50	2.0	Solvent free	97
2	**2**	50	2.0	Solvent free	85
3	**1**	50	2.0	MeOH	78
4	**1**	50	2.0	CHCl_3_	67
5	**1**	50	2.0	CH_3_CN	57
6	**1**	50	2.0	THF	81
7	**2**	50	2.0	MeOH	55
8	**2**	50	2.0	CHCl_3_	43
9	**2**	50	2.0	CH_3_CN	41
10	**2**	50	2.0	THF	76
11	**1**	50	1.0	Solvent free	52
12	**1**	50	5.0	Solvent free	98
13	**2**	50	1.0	Solvent free	30
14	**2**	50	5.0	Solvent free	85
15	**1**	25	2.0	Solvent free	32
16	**1**	40	2.0	Solvent free	81
17	**1**	60	2.0	Solvent free	96
18	**2**	25	2.0	Solvent free	25
19	**2**	40	2.0	Solvent free	80
20	**2**	60	2.0	Solvent free	84
21	Blank	50	-	Solvent free	10
22	H_2_L	50	2.0	Solvent free	18
23	Zn(NO_3_)_3_·6H_2_O	50	2.0	Solvent free	28
24	Cd(NO_3_)_3_·4H_2_O	50	2.0	Solvent free	23

^a^ Reaction conditions (unless stated otherwise): benzaldehyde (1 mmol), 2.0 mol% (relative to the aldehyde) of catalyst, trimethylsilyl cyanide (250 µL, 2 mmol), 1.5 h reaction time, at 50 °C. ^b^ Calculated by ^1^H-NMR as the number of moles of final product 2-phenyl-2-((trimethylsilyl)oxy)acetonitrile per mole of aldehyde X 100.

**Table 2:** Cyanosilylation of different substituted aldehydes with trimethylsilyl cyanide (TMSCN) with catalyst **1** or **2** ^a^.

Entry	Reactant	Yield ^b^ (%) with 1	Yield ^b^ (%) with 2
1	4-Nitrobenzaldehyde	>99	96
2	3-Nitrobenzaldehyde	98	95
3	4-Bromobenzaldehyde	>99	94
4	4-Chlorobenzaldehyde	95	89
5	4-Hydroxybenzaldehyde	91	75
6	3-Hydroxybenzaldehyde	88	67
7	4-Methoxybenzaldehyde	75	64
8	4-Methylbenzaldehyde	71	58

^a^ Reaction conditions: aldehyde (1 mmol), trimethylsilyl cyanide (250 µL, 2 mmol), 2.0 mol% of catalyst **1** or **2** for 1.5 h at 50 °C. ^b^ Calculated by ^1^H NMR.

**Table 3:** A comparison of catalytic activity of different reported coordination polymers in the cyanosilylation of aldehydes with trimethylsilyl cyanide (TMSCN).

Entry	Catalyst	Solvent/Temp/Time	Aldehyde	Yield (%)	Reference
1	**1**	Solvent free/50 °C/1.5 h	Benzaldehyde	97	This work
2	[Pr(3,5-DSB)(Phen)]_n_	Solvent free/50 °C/3 h	Benzaldehyde	78	[23]
3	[{Co_0.6_Ni_1.4_(H_2_O)_2_(Bpe)_2_}(V_4_O_12_)]·4H_2_O·Bpe	Solvent free/50 °C/16 h	Benzaldehyde	77	[24]
4	[NaCu(2,4-HPdc)(2,4-Pdc)]	Solvent free/50 °C/24 h	Benzaldehyde	85	[25]
5	[Sn(L1)_2_]	Solvent free/50 °C/4 h	Benzaldehyde	75	[26]
6	**1**	THF/50 °C/1.5 h	Benzaldehyde	81	This work
7	[Pb(L1)_2_]_2_	DCM/50 °C/6 h	Benzaldehyde	97	[27]
8	[Cu_3_(benzenetricarboxylate)_2_]_n_	pentane/40 °C/72 h	Benzaldehyde	57	[28]
9	[Cd_2_(1,4-NDC)_2_(DMF)_2_]_n_	Tolene/50 °C/72 h	4-Nitrobenzaldehyde	49	[29]
10	{[Zn(dpe)(μ-OOCCH_3_)_2_](H_2_O)}*_n_*	DCM/RT/24 h	Benzaldehyde	14	[30]
11	{[Zn_3_(4,4′-bpy)_4_(μ-O_2_CCH_2_CH_3_)_4_](ClO_4_)_2_(4,4′-bpy)_2_(H_2_O)_4_}_n_	DCM/RT/24 h	Benzaldehyde	22	[31]

3,5-DSB = 3,5-disulfobenzoic acid; Phen = 1,10-phenathroline; L1 = 3-amino-2-pyrazinecarboxylate; Bpe = 1,4-NDC = 1,4-naphthalenedicarboxylate; 2,4-H_2_Pdc = pyridine-2,4-dicarboxylic acid; dpe = 1,2-bis(4-pyridyl)ethene; 4,4′-bpy = 4,4′-bipyridine.

## Data Availability

Not applicable.

## Supplementary Materials

The following are available online, Figure S1: (A) π····π interactions (between pyrene rings) in coordination polymer 1, Figure S2: FT-IR spectra of catalysts 1 (A) and 2 (B) before (black) and after (red) the cyanosilylation reaction, Figure S3: PXRD spectra of catalysts 1 (A) and 2 (B) simulate (black), before (red) and after (blue) the cyanosilylation reaction, Figure S4: ^1^H-NMR spectra of solvent-free cyanosilylation of benzaldehyde with catalysts 1 (A) and 2 (B) in CDCl_3_ (entries 1 and 2, Table 1) (The protons are considered in the integrations are indicated in red colour), Table S1: Crystal data and structure refinement details for compounds 1-2, Table S2: Hydrogen bond geometry (Å, °) in compounds 1-2, Table S3: Selected bond distances (Å) and angles (°) for compounds 1-2.
