# Supplementary material for: Pyrene Carboxylate Ligand Based Coordination Polymers for Microwave-Assisted Solvent-Free Cyanosilylation of Aldehydes
Source: Molecules. 2021 Feb 19;26(4):1101. doi: 10.3390/molecules26041101 (PMC7922377; doi:10.3390/molecules26041101)
Supplement: Supplementary file 1 [file molecules-26-01101-s001.pdf]

molecules

MDPI

Supporting Information

Pyrene Carboxylate Ligand Based Coordination Polymers for Microwave-assisted Solvent-free Cyanosilylation of Aldehydes

Anirban Karmakar[1],* Anup Paul[1], Elia Pantanetti Sabatini[1], M. Fátima C. Guedes da Silva[1] and Armando J. L. Pombeiro[1,2]*

[1] Centro de Química Estrutural, Instituto Superior Técnico, Universidade de Lisboa, Av. Rovisco Pais, 1049–001, Lisbon, Portugal. E-mail: anirbanchem@gmail.com; pombeiro@tecnico.ulisboa.pt

[2] Peoples' Friendship University of Russia (RUDN University), 6 Miklukho-Maklaya Street, Moscow, 117198, Russian Federation.

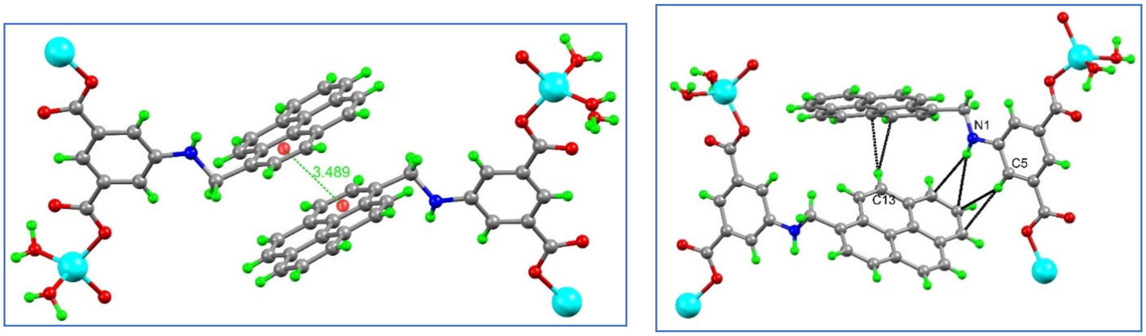

(**A**) (**B**)

Figure 1. (A) π····π interactions (between pyrene rings) in coordination polymer **1**. (B) N-H····π and C-H····π interactions in coordination polymer **1**.

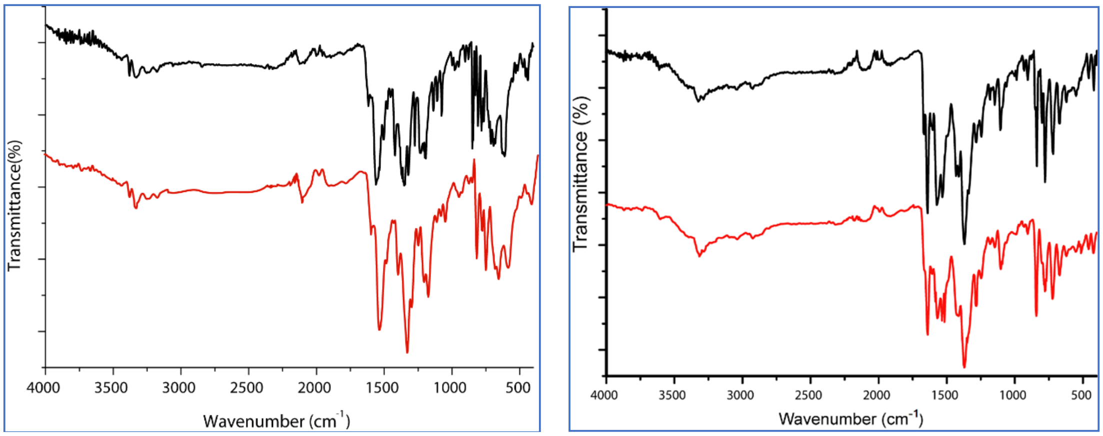

(**A**) (**B**)

Figure 2. FT-IR spectra of catalysts **1** (A) and **2** (B) before (black) and after (red) the cyanosilylation reaction.

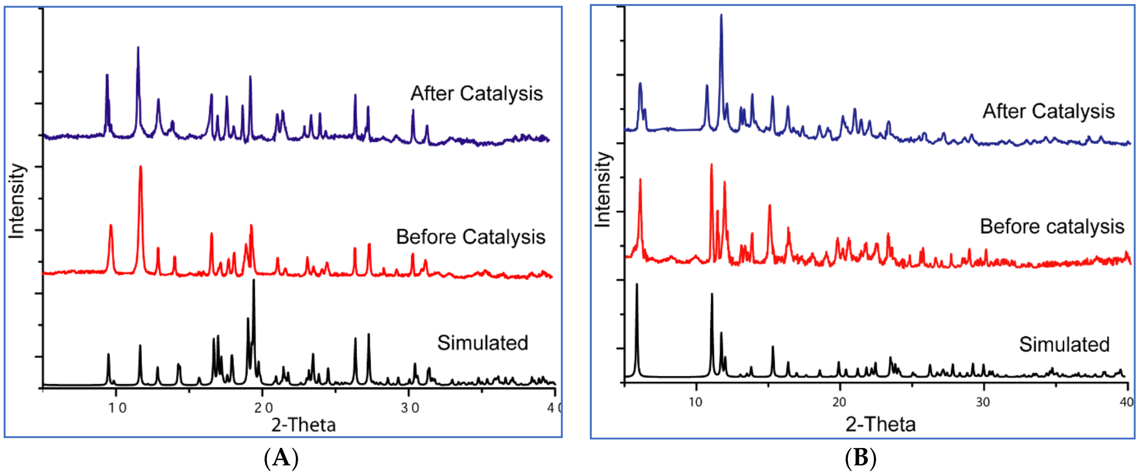

Figure 3. PXRD spectra of catalysts **1** (A) and **2** (B) simulate (black), before (red) and after (blue) the cyanosilylation reaction.

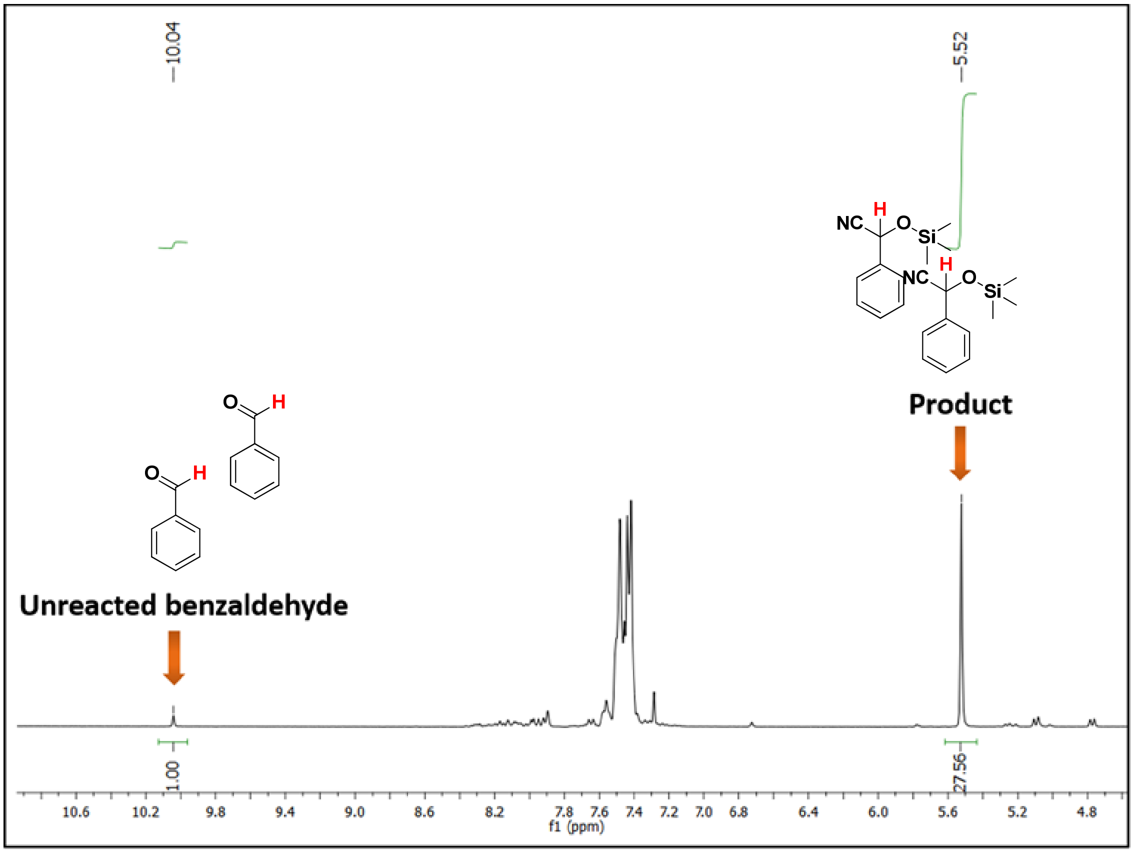

(**A**)

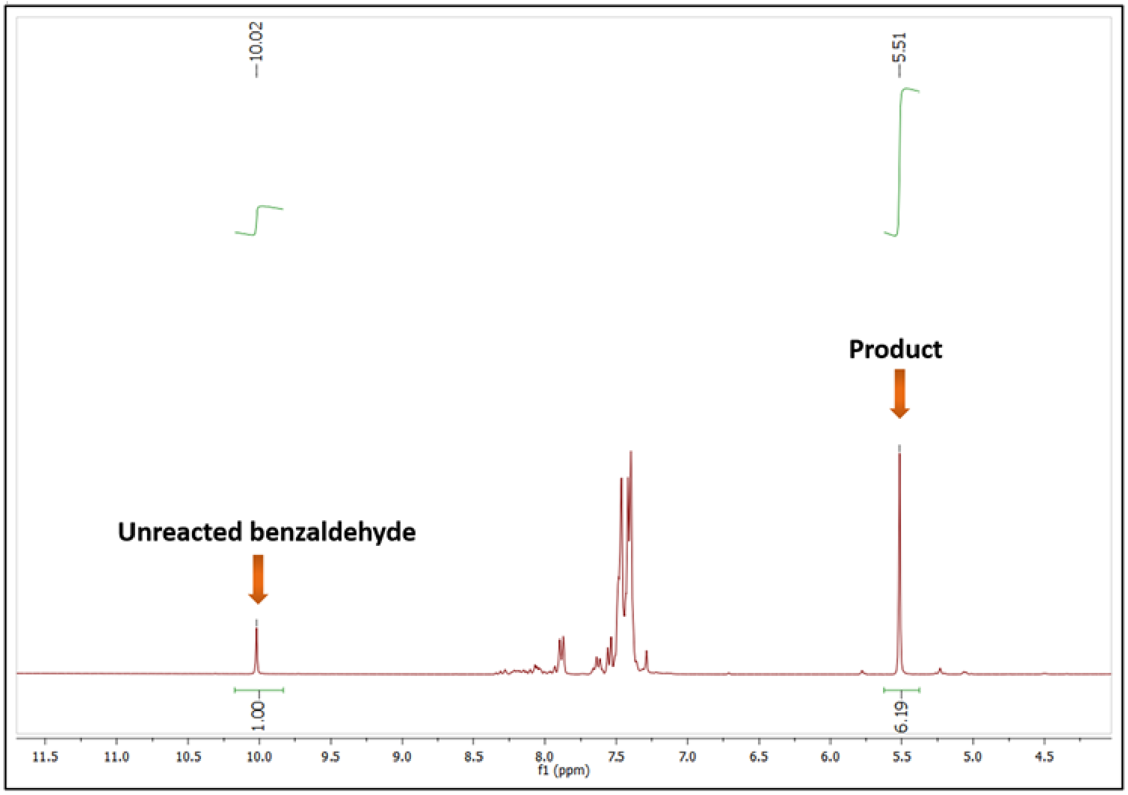

(**B**)

Figure 4. ^{1}H-NMR spectra of solvent-free cyanosilylation of benzaldehyde with catalysts **1** (A) and **2** (B) in $CDCl_3$ (entries 1 and 2, Table 1) (The protons are considered in the integrations are indicated in red colour).

Table 1. Crystal data and structure refinement details for compounds 1-2.

Identification name	1	2
Formula	$C_{25}H_{21}NO_7Zn$	$C_{25}H_{17}CdNO_6$
M.W. / g·mol^{-1}	512.80	539.79
Crystal System	Monoclinic	Triclinic
Space Group	P 21/c	P-1
Temperature / K	296	296
Wavelength / Å	0.71073	0.71073
a / Å	18.8023(11)	8.3311(7)
b / Å	10.2395(5)	8.3312(7)
c / Å	11.4154(7)	15.2829(12)
α / °	90	82.380(4)
β / °	97.744(2)	82.383(4)
γ / °	90	74.30
V / Å^3	2177.7(2)	1006.94(14)
Z	4	2
Density / Mgm^{-3}	1.564	1.780
Abs. Coeff. / mm^{-1}	1.177	1.131
F(000)	1056	540
Refl. collected	33290	25399
Refl. unique	5018	3889
Max. 2θ/°	27.552	26.048

Ranges (h, k, l)	-24<= h <=24<br>-13 <= k <=9<br>-14<= l <= 14	-10<= h <=10<br>-10 <= k <=10<br>-18<= l <= 18
Complete to 2θ (%)	99.8	99.8
Refl. with I > 2σ(I)	4077	3162
Data/Restraints/Parameters	5018/3/329	3889/3/310
Goof (F^2)	1.008	0.889
R1 [I > 2s(I)]	0.0410	0.0347
wR2 [I > 2s(I)]	0.1162	0.0856
R1 [all data]	0.0540	0.0534
wR2 [all data]	0.1244	0.0979

Table 2. Hydrogen bond geometry (Å, °) in compounds 1-2.

Compound	D-H···A	D···H (Å)	H···A (Å)	D···A (Å)	<D–H···A(°)
**1**	O5-H5B···O4	0.82	2.17	2.942(3)	157
	O6-H6A···O4	0.82	2.23	2.996(3)	155
	O6-H6B···O2	0.94	2.64	3.196(3)	118
	O7-H7B···O1	0.81	2.15	2.804(3)	138
	O7-H7A···O1	0.92	1.94	2.828(3)	164
	O5-H5A···O7	0.97	2.08	3.013(3)	159
**2**	O6-H6A···O2	0.91	1.96	2.851(5)	167
	O6-H6B···O4	0.90	1.86	2.748(4)	170
	N1-H1N···O2	0.88	2.27	3.033(4)	144
	C9-H9B···O3	0.97	2.65	3.139(5)	111

Table 3. Selected bond distances (Å) and angles (°) for compounds 1-2.

**1**	Zn1-O3 1.9700(16), Zn1-O2 2.0070(17), Zn1-O5 2.020(2), Zn1-O6 2.025(2).<br><br>O3-Zn1-O2 103.02(7); O3-Zn1-O5 111.33(8); O2-Zn1-O5 106.21(8); O3-Zn1-O6 109.16(9); O2-Zn1-O6 119.73(9); O5-Zn1-O6 107.32(10).
**2**	Cd1-O5 2.190(3), Cd1-O1 2.228(2), Cd1-O3 2.343(2), Cd1-O4 2.401(3), Cd1-O1 2.451(3), Cd1-N1 2.470(3).<br><br><O5-Cd1-O1 113.58(11), <O5-Cd1-O3 104.00(11), <O1-Cd1-O3 139.94(10), <O5-Cd1-O4 155.12(12), <O1-Cd1-O4 85.69(9), <O3-Cd1-O4 54.45(9), <O5-Cd1-O1 84.89(11), <O1-Cd1-O1 75.79(10), <O3-Cd1-O1 94.85(9), <O4-Cd1-O1 84.92(10), <O5-Cd1-N1 88.76(11), <O1-Cd1-N1 107.59(10), <O3-Cd1-N1 85.95(10), <O4-Cd1-N1 100.64(10), <O1-Cd1-N1 173.60(9).
